# Misdiagnosis in an Autistic Adolescent

**DOI:** 10.7759/cureus.68129

**Published:** 2024-08-29

**Authors:** Madison R Casey, Saveen Sall, Gina Parsons, Keerthiga Raveendran, Alexa Zaheri

**Affiliations:** 1 Department of Psychiatry, Louisiana State University Health Sciences Center Shreveport, Shreveport, USA

**Keywords:** neurodevelopmental disorder, autism, mental health disorder, misdiagnosis, autism spectrum disorder

## Abstract

With the increase in autism diagnoses in recent years due to improved public and clinical awareness, the association between autism and mental health has emerged as an important issue for patients and their caregivers. Although many with autism spectrum disorder also have coexisting mental health conditions, there exist differences in the presentation and etiology of these symptoms. This case report explains an interaction with a 17-year-old adolescent autistic male with a history of mild depression who was found non-responsive in the shower at home. Although the emergency medical team interpreted the scene as an attempted suicide, after lengthy interviews with the patient and the patient's family, the psychiatry team revealed a pre-existing condition, subdural empyema, that caused him seizures. This case highlights how autism characteristics can mask other relevant clinical details and delay proper diagnosis and treatment, especially when patients are non-responsive or exhibiting atypical behavior. It also underscores the importance of investigating all relevant clinical diagnoses, including those not related to psychiatric conditions. It is vital that healthcare providers learn how to effectively communicate with autistic patients to ensure proper treatment and improve patient outcomes.

## Introduction

Autism spectrum disorder (ASD) is a neurodevelopmental disorder affecting approximately 1.5% of individuals worldwide [1]. Since 2000, diagnoses of autism have increased due to improved awareness among the public and medical professionals [2]. Many mental health diagnoses can overlap with ASD, and approximately 70%-80% of autistic individuals also have a mental health diagnosis. Although the reasons for the high rates of psychiatric comorbidities with autism are unclear, difficulties in communicating with people with autism are a likely factor [3].

In one study, for example, participants with autism felt they received a mental health diagnosis more often than their non-autistic peers [4]. It is, therefore, important that the medical community be aware of and understand how autism can affect patient communication, care, and outcomes [5].

Autistic adolescent patients often present to emergency departments and acute care units in need of psychiatric evaluation and treatment [6]. These interactions have led to an increase in the rate of diagnosis of ASD in adolescents. These adolescents also are more likely than their typically developed peers to have repeated emergency department visits requiring psychiatric hospitalization and more likely to require outpatient care [6].

This difference is in part due to challenges in communicating with neurodivergent patients [7]. It is thus crucial that clinicians use supportive questioning when assessing autistic individuals, who may have deficits in social communication skills though they are capable of retaining similar amounts of information as their more typical peers [7]. Thus, according to the “task support hypothesis,” healthcare workers should allow ample time to ask supportive questions that may facilitate recall of episodic memories that can aid in diagnosis [7].

This case report describes an autistic adolescent patient who was brought to the emergency department, including how the psychiatric interview was conducted and areas requiring further investigation. The goal is to promote more effective communication and improve interview skills among clinicians assessing individuals with ASD, particularly in urgent and emergency care settings.

## Case presentation

A 17-year-old Caucasian male (hereafter, S.K.) with a history of autism, major depressive disorder, and attention deficit disorder came to the pediatric intensive care unit for consultation. He had been found non-responsive in the shower with a pillow and blanket and a bag containing various pills (trazodone, Zoloft, Adderall, and Ingrezza).

During S.K.’s initial evaluation by the weekend on-call psychiatry consult team, he was dressed in hospital-provided attire and lying in bed with no extrapyramidal symptoms or tremors. He was cooperative and maintained eye contact but appeared withdrawn with a flat affect. His mood was fine, but his speech rate and volume were decreased. He exhibited a vague thought process with no ruinations, rituals, or phobias and no persecutory, paranoid, or grandiose delusions. He was oriented to person and place but demonstrated impaired memory, poor attention, poor concentration, lack of insight, and faulty judgment. During the evaluation, he stated, “I overdosed on medications, sleeping medications.” When asked if he was trying to hurt himself, he responded, “I guess I was trying to kill myself, harm myself.” A few minutes later, he answered, “No,” further disclosing, “I have been having a hard time sleeping. My mind is always thinking of stuff and racing.”

When assessed for symptoms of depression, he responded that he was sleeping “too much” and that his appetite was “ok” and energy was “low.” He described his concentration as “poor” and denied previous psychiatric hospitalizations, any history of violence, previous suicide attempts, suicidal ideation, homicidal ideation, audiovisual hallucinations, and paranoia, as well as any alcohol, tobacco, illicit drug use, or criminal history. He also denied any history of physical, emotional, or sexual abuse. He stated that he was currently being treated by a psychiatrist and was prescribed Adderall and guanfacine for attention deficit disorder. He denied any occupational or employment history and was currently living with his mom, dad, and pet dog. The patient’s mother remained in the room and stated that her son is on the autism spectrum and was diagnosed with mild depression in eighth grade, for which he was prescribed Zoloft and Wellbutrin. He was currently enrolled as a high school senior in special education due to his autism diagnosis. She further disclosed that he had been acting more tired and sleeping more than usual, accompanied by a shift in mood. His family psychiatric history included the mother, who was diagnosed with bipolar disorder.

The weekend on-call psychiatry consult team recommended a Physician Emergency Certificate for legal status, which involuntarily detains a patient to a treatment center for 72 hours until a secondary exam can be done by the Coroner. In addition, the on-call team recommended that the patient’s primary team establish appropriate precautions for suicide, assault, elopement, seizure, and falls. Medications were deferred pending the results of a urine drug screening and ethanol labs. The psychiatry team also recommended obtaining a collateral history from the family and his support network as available. Obtaining collateral would be helpful in finding out details about the patient and the patient’s baseline behaviors that we otherwise would not be able to know.

The patient’s primary internal medicine team medically cleared him and thus the patient was eligible for admission to an inpatient psychiatric facility due to a suspected suicide attempt accompanied by a past history of mild depression. Upon discussing the case, the psychiatric team concluded it was unclear whether S.K. intentionally took the pills to harm himself. They agreed to reassess when his confusion cleared to determine the need for inpatient hospitalization for mood stabilization. His parents agreed with the need for inpatient treatment close to home. It was advised that the psychiatry team continue to follow the patient on the consult service until discharge to re-evaluate his mental status and mood and make further recommendations as needed. Further, it was recommended to provide the patient with appropriate supportive psycho-educational therapy about diagnoses, medications, compliance, and coping skills. The psychiatry team also discussed discharging him home if his mother arranged for outpatient mental health follow-up with their established provider shortly after discharge, with the caveat that he would be admitted to an inpatient psychiatric unit for stabilization if his provider recommended it. The patient’s provider was unable to be reached at the time.

His parents were advised to secure all medications at home as a safety precaution and to remove access to any guns or weapons. Close supervision at home was emphasized. All psychiatric medications were held until the patient’s behavior returned to his baseline. The patient was initially diagnosed with major depressive disorder recurrent severe without psychotic features along with his previous diagnoses of ASD and attention deficit hyperactivity disorder (ADHD). Attempts by the psychiatry team to obtain a collateral history from the patient’s outpatient psychiatrist were unsuccessful.

When the psychiatry team returned to evaluate the patient who had now been admitted to the hospital, he appeared delirious. The attending physician spoke in person to the parents in the patient’s room to gather more information. During the conversation, the parents stated that they did not think their son attempted to overdose on his medications. Rather, they thought that he may have been in the shower to relieve nausea caused by a worsening upper respiratory infection and then lost consciousness. He may have brought the bag of pills into the shower to take his scheduled dose of trazodone. No pills were missing from the pill bottles, and his mother denied recent stressors or behavioral changes in the patient. His urine drug screen also was negative for amphetamines or illicit substances.

The patient was then diagnosed with delirium and major depressive disorder (recurrent, in remission). The care team suspected his delirium could be caused by a worsening upper respiratory infection and the presence of seizures. In the absence of suicidal ideation and intentional overdose, discharge with outpatient provider follow-up was recommended. Psychiatric medications were held due to benzodiazepines worsening delirium. Medication for seizure control was recommended for use only when needed, rather than as scheduled for tics, to avoid worsening of mental status and delirium.

At S.K.'s reevaluation prior to discharge, he was alert and oriented to person, place, and time. He denied current depressive symptoms, suicidal ideations, recent stressors, and attempts to harm himself. He did not remember the events that led to admission, indicating he may have been altered due to medical issues. An MRI of the brain with and without contrast was ordered, revealing an intracranial empyema status post-evacuation and a small collection of apparent cerebral spinal fluid around the left frontal craniotomy bone flap and left maxillary sinusitis.

The patient was deemed not suicidal, homicidal, or gravely disabled and thus not eligible for inpatient psychiatric admission. The patient’s unconsciousness was more likely due to an intracranial abscess causing a seizure, not a suicide attempt. In the absence of safety concerns, the patient was medically and psychiatrically cleared for discharge with instructions to follow up with his outside mental health provider.

## Discussion

This case report demonstrates the importance of the clinician approach in psychiatric emergency cases involving children with ASD. A sensitive approach with an initially broad differential and emphasis on detailed collateral information from primary caregivers is crucial. Those with ASD have “persistent deficits with social communication and social interaction across multiple texts” as well as “restricted, repetitive patterns of behavior, interests, or activities” [8]. A positive healthcare experience for these patients requires good communication, clear explanations, and friendly attitudes of staff [9].

For S.K., the unfamiliar environment and interaction with new healthcare providers in an emergency setting likely exacerbated any difficulties he might typically have in answering questions about his emotions, mood, and feelings. Deviation from his usual routine also could have caused him to be more withdrawn. Therefore, the consultation-liaison psychiatry team tried to approach the patient in an especially sensitive way.

A noteworthy aspect of this case is its complex and mixed etiology: S.K. is an autistic child with comorbid depression who presented as delirious secondary to a medical illness and suspected suicide attempt. Conflicting information about the events leading to his emergency room visit (i.e., he initially stated that he was trying to harm himself and then stated this was not his intent), as well as symptoms such as low energy, low mood, fatigue, hypersomnia, and difficulty with concentration indicated depression, delirium, or other serious mental health conditions. A broader and more inclusive set of differential diagnoses and collateral history was needed to establish an appropriate diagnosis and clinical approach.

Studies on emergency visits among autistic children cite a higher prevalence of mental health diagnoses and suicidal ideation and attempts in this population, compared to non-autistic youth [6]. A 2022 study by Schott et al. analyzed data on a random sample of pediatric visits from 2008 to 2017 using the Nationwide Emergency Department sample, which contains data for over 30 million annual emergency visits [6]. They found that “the 10 most frequent primary diagnoses were physical conditions” but that children with developmental disabilities, autism, or attention deficit disorders were more likely to present with mood disorders, including suicidal or intentional self-harm, which was among the top 10 reasons for emergency visits for older children, “prompting 2.2% of visits in autistic children” [6]. The study concluded that compared to a random sample, children with autism are more likely to need emergency care due to psychiatric conditions, including self-harm, and thus “clinicians should treat these populations sensitively, recognize and assess the risk for self-harm, and facilitate continuing psychiatric care” [6]. A 2022 meta-analysis of 47 selected studies demonstrated that compared to the general population, autistic youth are at greater suicide risk, irrespective of age or sex: “The prevalence of suicidality of almost 40,000 autistic youth identified that one in four (25.2%) experiences suicidal ideation, and almost one in 10 (8.3%) attempt suicide” [10].

In our case study, the psychiatric consult team had to consider all three of these concerns: physical health conditions, mood disorders, and apparent suicidal or intentional self-harm behavior. The patient exhibited signs of delirium, such as providing conflicting information and exhibiting vague thought processes. Additional information collected from family and other sources helped clinicians understand that the patient was not at his baseline level of functioning during the initial emergency department evaluation. Without personal in-depth knowledge of a patient’s normal activities, behaviors, and cognitive abilities, it is crucial that clinicians be aware of symptoms and behaviors that warrant further investigation. After careful and thoughtful investigations, the clinicians in this case determined that a more likely cause of the patient’s symptoms and behavior was delirium secondary to underlying medical causes, rather than an attempt to self-harm.

Delirium is an acute confusional state characterized by a change in one’s baseline mental status. It is underrecognized and underdiagnosed in children and adolescents: neither the Diagnostic and Statistical Manual of Mental Disorders Fifth Addition nor the International Classification of Diseases 10/11 includes a definition of delirium specific to children and adolescents [11]. Yet, this condition has been “increasingly recognized and comprises 10% of all pediatric consultation-liaison referrals” [12]. Moreover, although delirium symptoms in adults and children share some features, many are more prominent in children, such as irritability, agitation, affective lability, sleep disturbances, and symptom fluctuations [12], many of which were observed in our case.

The literature offers little guidance on how autistic youth with comorbid mood disorders and delirium may present, compared to their non-autistic peers, and whether a significant difference in the time to correct diagnosis occurs between autistic and non-autistic youth. One study on youth presenting with agitation of mixed etiology recommended that emergency care clinicians use their “best judgment in assessing the relative contribution of each etiologic factor to the presentation” [13]. In the case of S.K., it was initially unclear that his atypical behavior was due to delirium and not to other contributing factors (e.g., ASD, co-morbid mood disorder, and suicidal ideation).

## Conclusions

Research has shown that autistic individuals are often misdiagnosed with other mental health diagnoses. It is vital that healthcare providers gain experience, exposure, and awareness of how to better interview, diagnose, and manage mental health in autistic patients. New interventions need to be introduced so that practitioners can better care for their patients, and further research is needed on delirium among pediatric patients, including children with neurodiverse conditions. This report contributes to the literature by offering guidance on how clinicians can assess the relative contributions of each etiologic factor in complex pediatric psychiatric cases, particularly those involving neurodiverse conditions.
